# Retraction: Optimizing planting geometry for barley-Egyptian clover intercropping system in semi-arid sub-tropical climate

**DOI:** 10.1371/journal.pone.0277626

**Published:** 2022-11-16

**Authors:** The PLOS ONE Editors

The *PLOS ONE* Editors retract this article [1] because it was identified as one of a series of submissions for which we have concerns about authorship, competing interests, and peer review. We regret that the issues were not addressed prior to the article’s publication.

MSS did not agree with retraction. MMM, AA, SF, KAK, SA, MIK, AH, MArif, MAhmad, and MT either did not reply directly or could not be reached. MIuH and SK responded but expressed neither agreement nor disagreement with the editorial decision.
